# Quantitative 3D imaging parameters improve prediction of hip osteoarthritis outcome

**DOI:** 10.1038/s41598-020-59977-2

**Published:** 2020-03-05

**Authors:** T. D. Turmezei, G. M. Treece, A. H. Gee, S. Sigurdsson, H. Jonsson, T. Aspelund, V. Gudnason, K. E. S. Poole

**Affiliations:** 1 Department of Radiology, Norfolk and Norwich University Hospital, Norwich, UK; 2 Cambridge University Engineering Department, Cambridge, UK; 3 Icelandic Heart Association, Kopavogur, Iceland; 4 Department of Rheumatology, Landspitalinn University Hospital, Reykjavik, Iceland; 5 Department of Medicine, University of Iceland, Reykjavik, Iceland; 6 Department of Medicine, University of Cambridge, Cambridge, UK

**Keywords:** Predictive markers, Osteoarthritis, Biomedical engineering, Statistics

## Abstract

Osteoarthritis is an increasingly important health problem for which the main treatment remains joint replacement. Therapy developments have been hampered by a lack of biomarkers that can reliably predict disease, while 2D radiographs interpreted by human observers are still the gold standard for clinical trial imaging assessment. We propose a 3D approach using computed tomography—a fast, readily available clinical technique—that can be applied in the assessment of osteoarthritis using a new quantitative 3D analysis technique called joint space mapping (JSM). We demonstrate the application of JSM at the hip in 263 healthy older adults from the AGES-Reykjavík cohort, examining relationships between 3D joint space width, 3D joint shape, and future joint replacement. Using JSM, statistical shape modelling, and statistical parametric mapping, we show an 18% improvement in prediction of joint replacement using 3D metrics combined with radiographic Kellgren & Lawrence grade (AUC 0.86) over the existing 2D FDA-approved gold standard of minimum 2D joint space width (AUC 0.73). We also show that assessment of joint asymmetry can reveal significant differences between individuals destined for joint replacement versus controls at regions of the joint that are not captured by radiographs. This technique is immediately implementable with standard imaging technologies.

## Introduction

Hip osteoarthritis is an enormous health burden estimated to affect one in four individuals in the USA during their lifetime^1^. The American Joint Replacement Registry reported 280,000 primary total hip replacements (THRs) performed in 2017 from 854 participating facilities^2^, with 92,000 THRs registered in the UK for the same year^3^. These are among the most common surgical procedures performed and remain the mainstay of treatment for end-stage osteoarthritis.

There are no approved therapies for prevention of osteoarthritis or reversal of progression. A lack of effective biomarkers that might facilitate therapeutic clinical trial successes has previously been identified as a serious limitation and remains an ongoing challenge^4^. Until recently, the US Food and Drug Administration (FDA) only approved 2D radiographic joint space assessment for clinical trial imaging endpoints^5^, although it appears that these guidelines will be relaxed to include cross-sectional imaging with the aim of facilitating therapy development^6^. This is an opportunity for a change in approach to imaging assessment of joints, particularly in how to identify relevant structural changes as they fail.

Historically the OARSI-OMERACT (Osteoarthritis Research Society International–Outcome Measures in Rheumatology) defined relevant radiological progression at both the hip and knee as progressive loss of joint space width (JSW)^7^. Clinical and epidemiological studies define disease with radiographic Kellgren and Lawrence (KL) grading^8^, but the KL system has been shown to suffer from variable interpretation^9^. However questions have been raised over the link between symptomatic hip osteoarthritis and radiographic findings, with concerns that clinical cases of hip osteoarthritis might be missed if radiographs alone were relied on for diagnosis^10^.

In the clinical environment, the traditional American College of Rheumatology (ACR) classification criteria for hip osteoarthritis allow for the inclusion of radiographic joint space narrowing. These were under peer review at the time of publication^11,12^. In the UK, the National Institute for Health and Care Excellence (NICE) guidelines from 2014 do not recommended the use of imaging in the diagnosis of osteoarthritis^13^. Yet the role of radiographic imaging in stratification and assessment of disease progression for research studies and clinical trials has remained unchallenged.

Assessment of radiographs and, in most circumstances, MRI relies on 2D images being interpreted by human observers to give a semiquantitative score or grade of disease: for example the KL system grades from 0 to 4 (equating to none-possible-mild-moderate-severe)^8^. Beyond morphological cartilage thickness measurement^14^, other quantitative MRI techniques such as compositional T2, T1rho, ultrashort echo and dGEMRIC (delayed gadolinium enhanced MRI of cartilage) time measurements have been developed, but their role in prognostication is yet to be clearly defined^15,16^. Semiquantitative MRI scoring systems have also been developed for the assessment of disease at the hip (HOAMS) and knee (MOAKS)^17,18^. No single MRI technique has come to the fore as an effective predictor of clinically relevant disease states or outcomes.

Given the lack of success so far in finding a solution, our idea was to take a step back from MRI towards the current standard of 2D radiographic JSW measurement, then look for a direct improvement on this.

CT, like radiography, is an x-ray based clinical imaging technique, but unlike radiography it has the ability to create large and accurate 3D imaging data volumes. Already an essential clinical technique, CT also plays an important role in osteoarthritis research and has been implemented in epidemiological cohort studies (the Multicenter Osteoarthritis Study) using dual energy and standing knee CT, and with low dose whole body CT (the APPROACH study). CT has been mainly used for investigating diagnostic potential, but not yet disease monitoring or prognostication^19^. Our aim has been to develop an image analysis technique that uses clinical CT imaging to reveal the distribution of JSW in 3D, believing that it could outperform current gold standard 2D imaging approaches, while accepting this is likely to come at the cost of increased radiation dose.

The joint space is essentially laminar and, in some situations, thinner than the resolution of the CT data we would like to use to measure it. Imaging system blur, also known as point spread function (PSF), sets the limit below which features cannot be measured accurately. In CT this is often approximated as a simple function with a Gaussian profile. Modern CT systems can be optimised to improve image resolution, but substantial gains can only be achieved at the cost of increased noise unless radiation dose is also increased, which is undesirable. JSW distances at relatively narrower joints such as the hip or ankle (compared to the knee), or with more advanced degenerative joint disease at any joint, can be below this threshold.

In order to achieve accurate measurements of JSW from CT imaging, we remove this blur using a constrained deconvolution approach, where the constraint—here prior knowledge of bone density around the joint space—acts to limit noise in measurement while still improving accuracy. We call this approach joint space mapping (JSM). This process is repeated at multiple locations each in a different direction, to yield quantitative JSW measurements in 3D. It has been recently validated as accurate and reliable, with twice the reported reliability of radiographic 2D minimum JSW measurement^20^.

**Figure 1:**
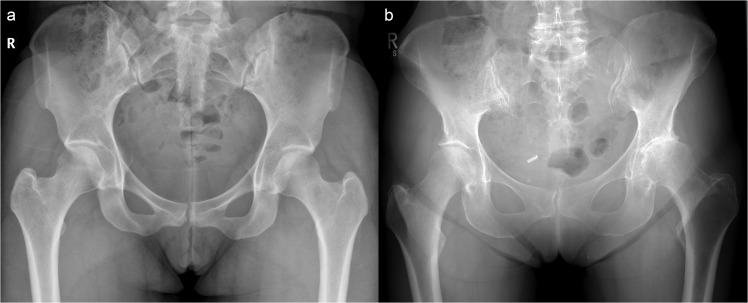
Anteroposterior radiographs of the pelvis in two adult females. The individual in (**a**) has no evidence of radiological osteoarthritis on either side (KL grade 0); however, the individual in (**b**) has marked disease asymmetry, with no radiological disease in the right hip (KL grade 0), but moderate disease in the left (KL grade 3).

The ability to map JSW in 3D also delivers an opportunity to revisit a rarely considered phenomenon. Experience from our clinical practice has shown that one hip tends to degenerate prior to the opposite, as demonstrated in Fig. 1. To the best of our knowledge, only two previous studies have specifically commented on radiographic JSW asymmetry^21,22^. The first, by Reis *et al*. in 1999, looked at the difference in superior JSW in 171 individuals without hip pain or radiographic evidence of disease, concluding that side differences in JSW greater than 0.7 mm at the superior joint space strongly suggested pathological joint narrowing. In 2004, Lequesne *et al*. then examined the same superior locations for radiographic minimum JSW and concluded that asymmetry was rare, seeing a difference greater than their limits of agreement (1.45 mm) in only 13 out of 221 subjects (5.9%)^22^. Using the opposite hip could provide an approach for a single time-point assessment of osteoarthritis risk from this internal comparison.

In this paper we describe the JSM process and its first application in a clinical cohort of healthy older adults using 3D statistical analysis. Section 2 describes the technical details of JSM, previously introduced in the validation study published in 2018, but because they are fundamental to the technique warrant revisiting here^20^. Section 3 describes the clinical cohort and CT imaging on which JSM and subsequent 3D statistical analyses were performed. Section 4 then uses a statistical shape model (SSM—a by-product of the JSM process) to look at 3D shape variation across the study cohort, and the relationships of 3D shape modes with clinical outcome of future THR. Section 5 examines the dependence of 3D JSW on these same factors using statistical parametric mapping (SPM). In this section we explore two specific hypotheses: (1) 3D JSW measurement would be significantly different in cases with future THR compared to controls; and (2) the same would be the case for the side-side difference in 3D JSW as a measure of joint asymmetry. In section 6 we apply a predictive model for future THR using parameters derived from these analyses, with the hypothesis that quantitative 3D image analysis would perform better than current standard 2D measures. Finally, in section 7, we discuss these results and their implications for imaging approaches to structural joint disease.

## Joint Space Mapping (JSM)

**Figure 2:**
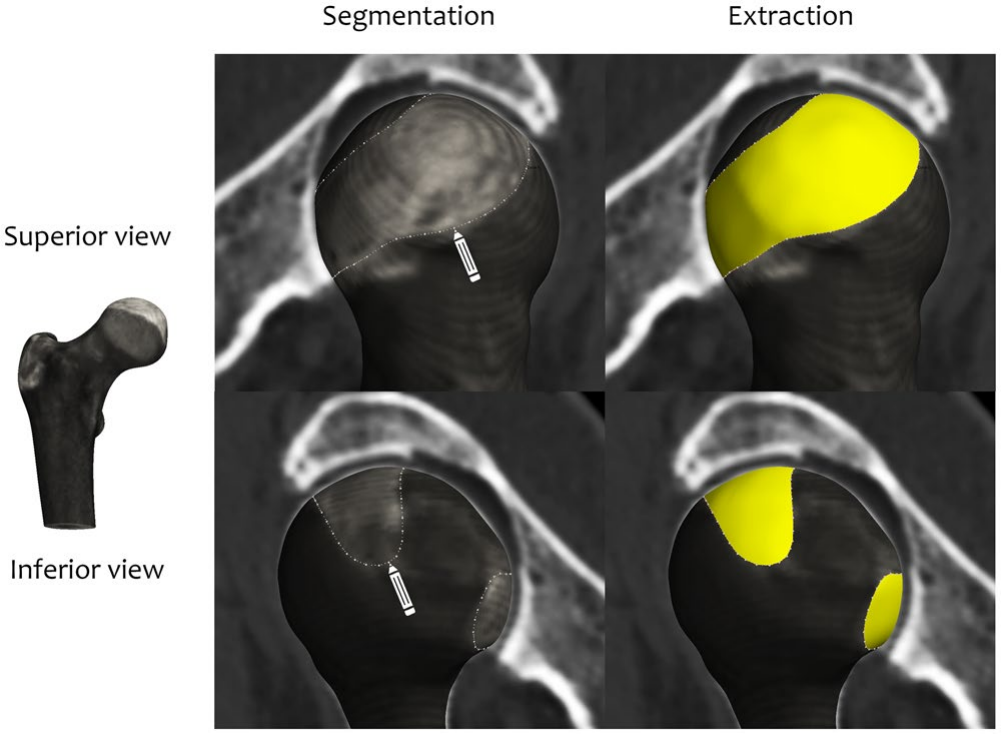
Segmenting and extracting the 3D joint space patch (yellow) around the perimeter of the shadow of the acetabulum projected back onto the 3D proximal femoral object surface (from Turmezei *et al*.)^20^. Images are from the Stradwin software (http://mi.eng.cam.ac.uk/~rwp/stradwin/).

Joint space mapping analysis uses standard clinical CT imaging data to create maps of joint space width distribution in 3D. The first step is to segment the proximal femur from cross-sectional (axial) CT data, from which a triangulated mesh is constructed using regularised marching tetrahedra^23^. We employ a semi-automatic manual segmentation approach, which can be guided by a shape model, however segmentations can also be performed with any appropriate manual, semi-automatic, or automatic technique. 3D joint space patch segmentation is then performed for each hip joint: the perimeter of the joint space is set by the shadow of the acetabulum projected back onto the 3D proximal femoral surface (Fig. 2).

**Figure 3:**
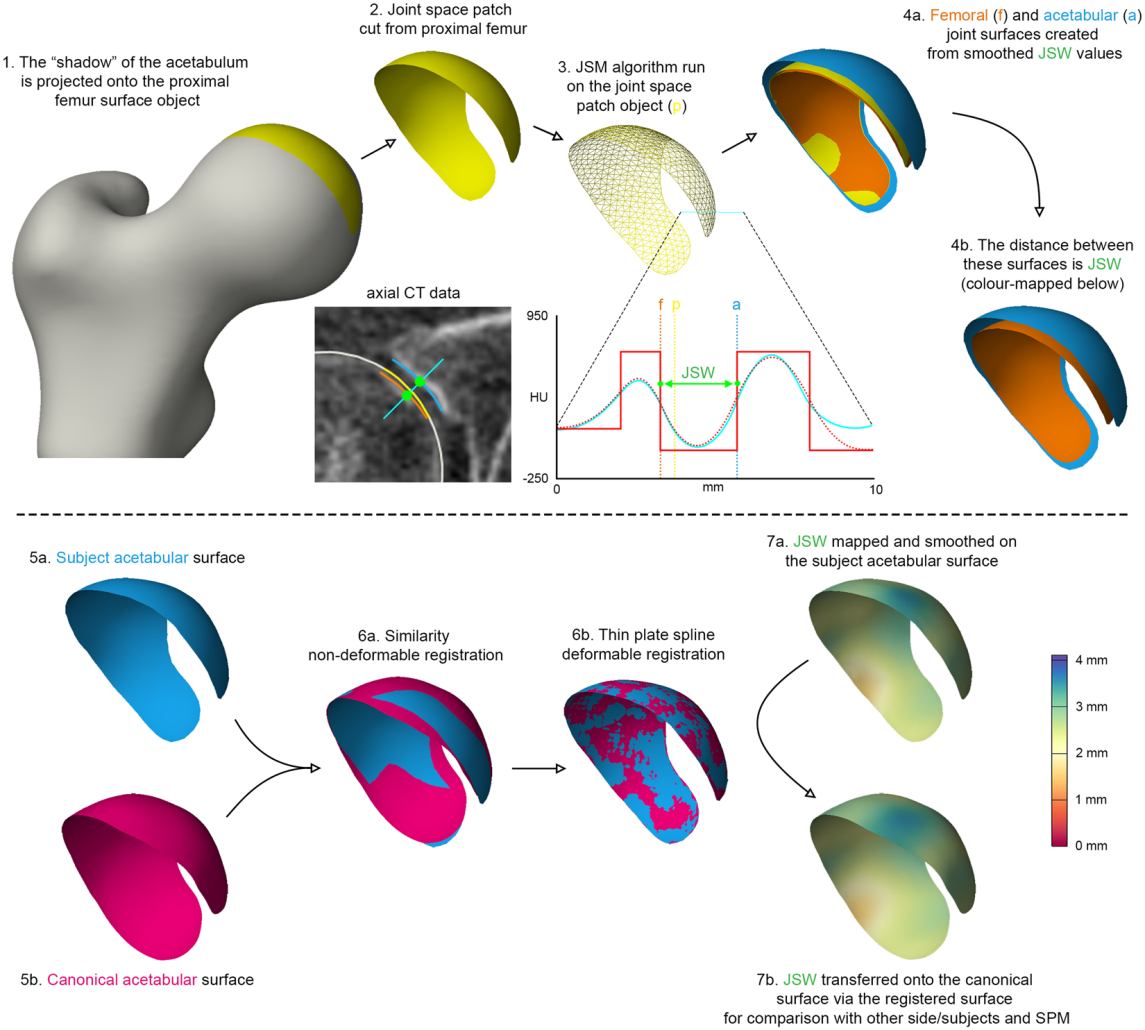
The joint space mapping pathway (1–4), which requires registration and data transfer to a canonical acetabular surface (steps 5 to 7) for analysis with statistical parametric mapping and statistical shape modelling. 3D vectors at each vertex from the registration of the canonical surface to the subject surface (Step 6) are used as the input for principal component analysis.

Each vertex in the joint space patch (yellow in Fig. 3, steps 1 to 4), is a measurement point at which a 1D sample line is passed through the 3D imaging data volume at a normal to the object surface (Fig. 3, step 3). The average number of vertices in a joint space patch in the clinical study we describe in this paper is ~2,000, ranging from 1,250 to 3,500 depending on the size and extent of the acetabulum. A Gaussian function is fitted to the interpolated imaging data along this sample line (solid red curve fitted to the cyan curve in step 3, Fig. 3), using an optimiser. This optimiser presumes a fixed cortical density for each joint space patch (the horizontal tops of the solid red step functions in the same graph). Other parameters of the Gaussian function, including the location of the subchondral bone edges at the joint space (the vertical lines of the solid red line step functions) are determined by the optimiser. This deconvolution approach to the 3D measurement of a laminar structure in CT imaging data has been described by Treece *et al*.^24^. The distances between outer subchondral bone layers at the joint is taken as JSW (Fig. 3, steps 3 & 4). Independent JSW measurements for each vertex in the original joint space patch are smoothed to reduce noise and cover any missing measurements due to occasional failure of the optimiser to converge on a solution. The subsequent measurements at every vertex can be used to generate new 3D joint surfaces which precisely locate the subchondral bone edges. All these steps are performed using Stradwin, software freely available to download at http://mi.eng.cam.ac.uk/~rwp/stradwin/.

Technical validation for the accuracy, precision, and reproducibility of this technique has been previously reported^20^. In contrast to the originally published technique, density estimation in this paper is performed on the joint space patch rather than the whole proximal femur. This preserves the concept of estimating density from the object on which measurements are performed, giving an estimate of peak cortical density from subchondral bone local to the joint rather than from femoral cortex at more distant trochanteric and subtrochanteric regions^24^. A comparison of JSW values from both techniques showed that across the total of ~1.1 million measurement points, there was a systematic overall underestimation of only 0.2 mm compared to the high resolution CT gold standard from Turmezei *et al*. in 2018 (compared to an overestimation of 0.1 mm in that study)^20^.

As shown in Fig. 3 (steps 5 to 7), data output from JSM is fundamentally surface-based. SPM is an appropriate analysis tool for such data, but each individual joint space first needs to be mapped to the same canonical 3D acetabular joint surface, shown in relation to a canonical proximal femur in Fig. S1. This surface contains approximately 2,300 vertices and was created from the 20 test subjects in the JSM validation study^20^. It is registered to all individual acetabular surfaces in turn using a similarity transformation with an iterative closest point (ICP) registration algorithm. This is followed by a thin plate spline transformation that includes matching the rims of the two objects (Fig. 3, steps 5 & 6)^25^. JSW measurements are transferred from each individual acetabular surface to the canonical surface at closest neighbouring vertices, then smoothed further as required for SPM analysis. These steps are performed in wxRegSurf, software freely available at http://mi.eng.cam.ac.uk/~ahg/wxRegSurf/.

For side difference 3D JSW maps, data mapped onto the canonical surface from left and right hips in the same individual are subtracted from each other: the replaced hip minus the opposite side; random side selection if both replaced; and according to case side-matching by random selection in controls.

## Clinical Cohort Study

### Study groups

We undertook a nested case-control study within the prospective AGES-Reykjavik cohort of 3,133 healthy older adults, the design and recruitment of which have been described in detail^26^. After initial 2:1 matching of all THR cases for age and gender, exclusion criteria were: THR for fracture rather than osteoarthritis (16 hips); movement artefact (6 hips); incomplete joint coverage (2 hips); and joint ankylosis (1 hip). This gave 80 case individuals with a THR performed on at least one side in the subsequent 5 years and 187 controls who had neither hip replaced in the same period. 2 hips in each group were unpaired, leaving 78 case individuals and 185 control individuals with both hips for subsequent SPM analysis of both standard 3D JSW and side difference in 3D JSW—see Table 1. 17 individuals in the THR case group had both hips replaced within the 5-year follow-up period. Hip pain (HP) at study baseline was recorded if there was any pain in either hip for more than one continuous month in the last year. From all 263 individuals with imaging of both hips, 53 had hip pain at baseline (14 on both sides), while 210 individuals had no pain.

**Table 1:** Demographics of the analysis groups.

		n hips	n ind.	Age (yrs)	Sex	Ht (cm)	Wt (kg)	BMI (kg/m^2^)
ALL HIPS	Controls	433*	187	74.3 ± 4.9	164 M 282 F	167.6 ± 9.2	77.5 ± 13.5	27.6 ± 4.2
	THR cases	97	80	74.4 ± 4.9	35 M 62 F	167.0 ± 8.5	77.5 ± 12.6	27.8 ± 4.2
	p value	—	—	0.88	0.90	0.58	0.98	0.62
PAIRED HIPS	Controls	—	185	74.3 ± 4.9	68 M 117 F	167.6 ± 9.3	77.5 ± 13.7	27.5 ± 4.2
	THR cases	—	78	74.3 ± 4.7	28 M 50 F	167.9 ± 8.8	77.7 ± 12.9	27.9 ± 4.4
	p value	—	—	0.92	0.89	0.58	0.91	0.52

There were no significant group-wise relationships (p < 0.05). *This number includes the non-THR hips from the THR case individuals.

There were no significant differences between THR cases and non-THR controls according to age at the time of imaging and study baseline BMI according to a 2-tailed t-test, or sex according to the chi-squared test (p > 0.05 in all cases). All 530 available hips were used in 3D SSM analysis (see Table 1).

### CT imaging acquisition

All CT imaging was acquired helically in the supine position at study baseline using a 4-detector clinical CT system (Sensation 4, Siemens Medical Systems, Erlangen, Germany) with the following parameters: 120 kVp, 140 mAs, 1 mm slice thickness, pitch of 1, pixel size 0.977 mm, matrix size 512 × 512. Hip positioning was not controlled at the time of acquisition. The study imaging protocol was to scan a range from 10 mm superior to the acetabulum to at least 3 mm inferior to the lesser trochanter (2 individuals were excluded from failure of adherence to this imaging protocol, as above). The same proprietary smooth image reconstruction kernel was automatically applied to all acquisitions.

The approximate dose for an examination was ~2.5 mSv, with a mean ± SD dose length product (DLP) across the cohort of 280.15 ± 27 mGy.cm and weighted CT dose index (CTDI_w_) of 21.6 ± 0 mGy. According to the guidelines for radiation protection from the International Commission on Radiological Protection (ICRP Publication 62, Radiological Protection in Biomedical Research, Annals of the ICRP 22 (3) 1991), the risk of biological damage due to ionizing radiation is considered to decrease with age, such that for participants age 70–80 years is 1/5^th^ to 1/10^th^ lower than it would be for participants aged 50 years, noting that the mean age of the participants in this study was 74.3 ± 4.9 yrs.

### Radiographic disease scoring

KL grade and then minimum 2D joint space width (JSW) had been previously recorded for each hip by a single blinded observer as part of a separate study in 2014 with a method digitally reconstructing radiographs from the CT data^27^. The distributions for these scores across all hips from the study are shown in Fig. S2 and Table S1.

### Ethical approval and informed consent

The AGES-Reykjavik study was approved by the Icelandic National Bioethics Committee, (VSN: 00–063), which acts as the Institutional Review Board for the Icelandic Heart Association, and from the Data Protection Authority. Approval for the current study was received from the AGES-Reykjavik executive committee on 28th August 2015. Written informed consent for use of data had previously been obtained from all participants. All methods were performed in accordance with the relevant guidelines and regulations.

## Statistical Shape Modelling

Joint space mapping creates sets of vertices for corresponding 3D femoral and acetabular surfaces at each hip (orange and blue surfaces respectively in Fig. 3), each vertex pair separated by a vector with the magnitude of JSW and a direction perpendicular to the original joint space patch surface cut from the proximal femur. As each surface is a triangulated mesh, the registration of the canonical surface to each individual acetabular surface (Fig. 3, steps 5 & 6) generates a 3D vector for each vertex as it is displaced towards the other surface. We then use principal component analysis (PCA) to determine orthogonal modes of shape variation from the average acetabular surfaces in the cohort^28^.

Horn’s parallel analysis showed that the first 16 shape modes were greater than noise in the PCA coefficient matrix (Fig. S3)^29^, while the first 7 shape modes accounted for up to 90% of overall shape variation.

In order to control for the clustering effect of two hips coming from one individual, generalised estimating equations (GEEs)^30^ were used to calculate odds ratios (ORs) for association of shape modes with future THR, including age and BMI in the model; sex was not included because of its strong association with shape mode 1 (r^2^ = 0.70) and the risk of multicollinearity, but its effect is therefore represented by this mode. OR values were calculated for each SD increment in shape mode coefficient, with the SD value adjusted according to the equivalent number of paired hips in the analysis as weighted by 1/(1 + R), where R is the correlation between shape mode coefficients from the two sides in the same individual (Table 2)^31^. The threshold for significance values was reduced from p < 0.05 using a Bonferroni correction factor of 18 (16 shape modes, plus age and BMI), making p < 0.003 the conservative significance threshold.

**Table 2:** Odds ratios (ORs) with ±1.96 × SE (standard error) limits for future THR.

	OR (±1.96 × SE limits)	p value	Description
Age	0.99 (0.93–1.05)	0.81	—
BMI	0.98 (0.91–1.04)	0.44	—
Shape mode 1	**1**.**43** (**1**.**16**–**1.77**)	**0**.0001*	*Scale*
Shape mode 2	1.75 (1.40–2.20)	**0**.00001*	*Acetabular expanse from central to outer joint*
Shape mode 3	1.37 (1.15–1.63)	0.0098	*Squatness to the anterior aspect of the joint*
Shape mode 4	1.02 (0.86–1.20)	0.88	*Anterior and posterior joint limb convergence*
Shape mode 5	0.88 (0.71–1.09)	0.28	*Acetabular depth/shallowness*
Shape mode 6	1.39 (1.15–1.68)	**0**.001*	*Anterior-posterior skew*
Shape mode 7	1.18 (0.98–1.40)	0.13	*Width across the anterosuperior joint space*

ORs for shape mode are for one standard deviation increment in mode coefficients. Significant results below the conservative Bonferroni corrected threshold of p < 0.003 are emboldened and marked with*. No shape mode beyond 7 met significance, with these results presented in Table S2.

**Figure 4:**
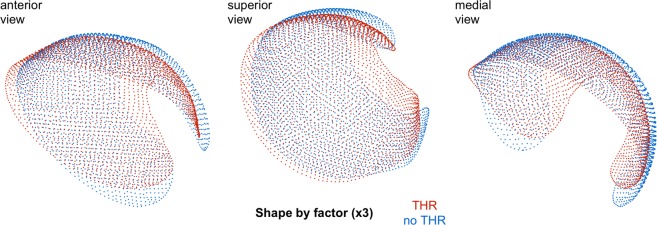
The average shape of future THR hips compared to non-THR cases using all shape modes. Each point in the plots is a vertex displaced from the canonical model by a vector representing the mean displacement for the given study group (THR cases in red, controls in blue). The vector magnitudes have been increased by a factor of 3 to emphasise differences. See supplementary material for a dynamic 3D visualisation.

We present the first 7 shape mode as 3D point clouds ±3 SD from the mean cohort shape (Fig. S4), as well as showing the mean shapes for future THR cases compared to controls (Fig. 4).

These 3D SSM results suggest that a smaller joint space (shape mode 1 and, by correlation, also being female), increased acetabular coverage medially and laterally (shape mode 2), and a skew in the anterior and posterior joint space limbs (shape mode 6) pose an increased risk for future THR. However, caution is required in the interpretation of these results, since registration of these objects are somewhat arbitrary due to the ill-posed object matching problem, with different registrations potentially capable of delivering different results. We chose what we believe is the current best approach.

## Statistical Parametric Mapping

Statistical parametric mapping (SPM) was performed on all 263 individuals with imaging of both hips to look for regions of 3D JSW and its side difference that might be significantly different between THR cases and the control group. SPM is a 3D statistical analysis technique initially developed for application in neuroimaging to allow regionally located statistical inferences to be made from the comparison of data at multiple points. SPM uses a general linear model (GLM) at every point on the surface to account for variability in measurement data in terms of experimental and confounding factors^32^. Here we apply it to surface distributed JSW data in a similar fashion to previous studies that have looked at the relationship between cortical bone parameters and hip fracture type^33^. 3D JSW maps and shape mode coefficients, for both hips from the same subject, whether in bilateral THR cases or the control group, were averaged to account for cluster bias from within-subject correlation.

The SPM general linear model included THR outcome as the experimental effect and shape modes (SMs) 1 to 7 as confounding effects, given that they make up to 90% of overall shape variation (Eq. 1). This approach is applied to account for systematic registration bias that might alter significance of results^34^.

1 $$\begin{array}{rcl}{\rm{JSW}} & = & 1+{\rm{THR}}+{\rm{SM}}1+{\rm{SM}}2+{\rm{SM}}3+\,{\rm{SM}}4+{\rm{SM}}5+{\rm{SM}}6+{\rm{SM}}7\end{array}$$

Age and BMI did not have any effect on results and were therefore not included in this model. Sex was removed to avoid multicollinearity because of its correlation with shape mode 1 (r^2^ = 0.7), but in any case did not have an effect on results when tested. No other covariate correlation was above 0.4. Standard two-tail F testing across the cohort gave p-values at each measurement location that were uncorrected for multiple comparisons. As required for SPM, random field theory furnished p-values corrected for multiple comparisons to control for false positive results (type I errors)^32^. Significant result clusters are shown as unmasked region of interests (ROIs) over the mean difference between THR cases and controls, each of which are mapped onto the canonical acetabular surface in 3D. This SPM analysis was performed in MATLAB 2018a (© 1984–2018, The MathsWork, Inc.) using the Surfstat package^35^.

**Figure 5:**
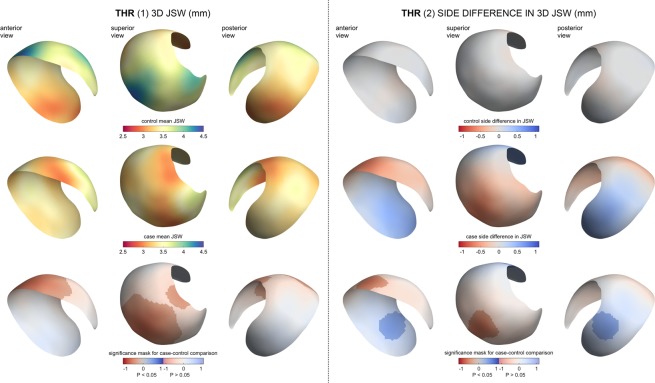
SPM results from the THR experiments. The left side of the plot shows (1) standard 3D JSW measurement; the right side of the plot shows (2) side difference in 3D JSW.

SPM revealed a large ROI across the superior joint space in which 3D JSW was significantly dependent on future THR (p < 0.05), narrower in THR cases compared to controls by up to 1 mm (Fig. 5, left, bottom row). Although non-significant, there was a trend for the joint space to be wider by up to ~0.5 mm posteriorly, suggesting that the femoral head migrates anterosuperiorly in the acetabulum as the joint fails. This zone of SPM significance was used as the ROI from across which a minimum 3D JSW value was taken forward to the prediction model, divided by the global mean 3D JSW to deliver a single parameter (min3D, Section 6). A smaller significant region was identified for side difference in 3D JSW across the superior joint space of up to 1 mm asymmetry (Fig. 5, right, bottom row), but also with a region significantly wider by up to ~0.75 mm in the posterior joint space, again suggesting that there is a tendency for failing hips to migrate anterosuperiorly.

## ROC Analysis for Future THR Prediction

Receiver operating characteristic (ROC) curves with area under the curve (AUC) values were calculated using a leave-one-out cross-validation of a predictive model for future THR. This model used baseline hip pain (HP), KL grade, minimum 2D JSW (min2D), minimum 3D JSW within the SPM significance region of interest (ROI) divided by mean global 3D JSW (min3D), and the first 7 shape mode coefficients (SM) accounting for 90% of overall shape variation. This analysis was also performed in MATLAB 2018a (© 1984–2018, The MathsWork, Inc.)

**Figure 6:**
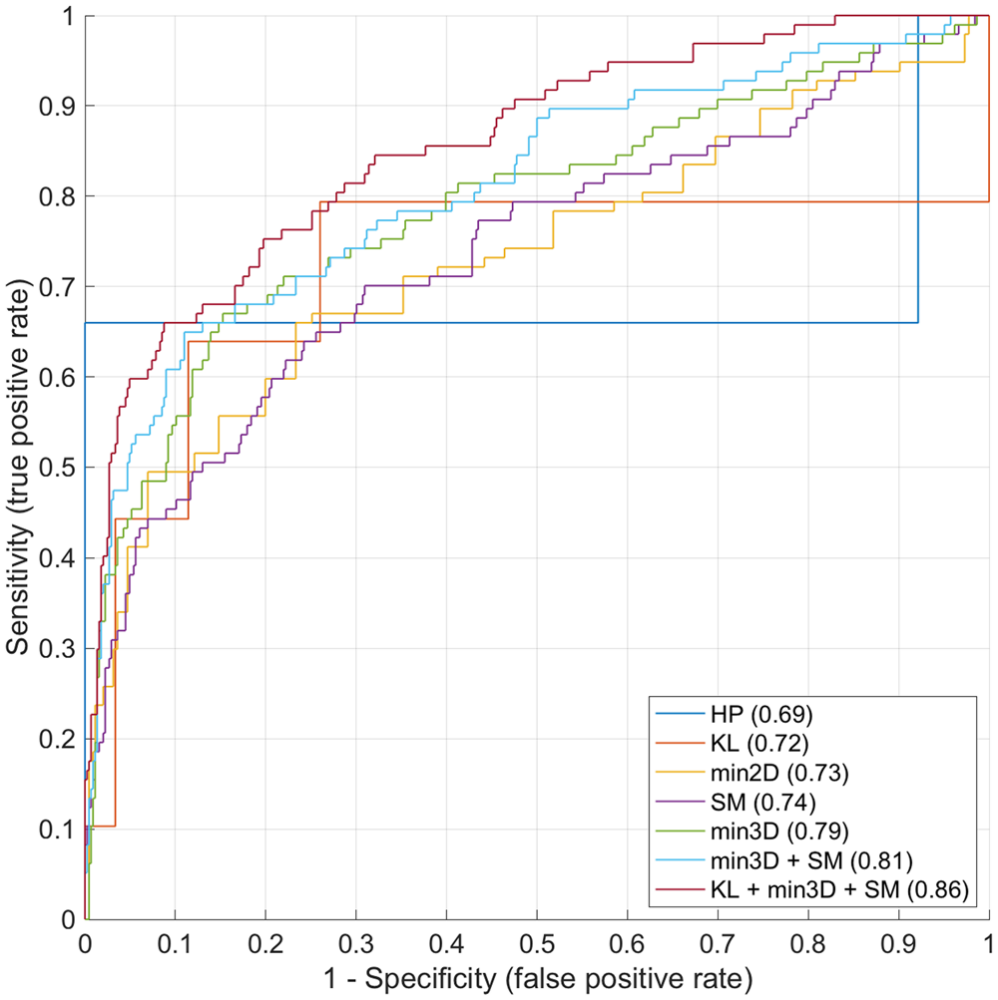
ROC curves with AUC values for each of the predictive models for future THR.

This predictive model for future THR showed that hip pain was the poorest predictor (AUC = 0.69). KL grade (0.72), minimum 2D JSW (0.73) and the first 7 shape mode coefficients (0.74) were all similar, while there was a slight improvement for minimum ROI 3D JSW relative to the global mean (0.79). AUC increased for combined 3D JSW and shape mode data (0.81) and was further improved when KL grade was also included (0.86), an 18% improvement on the FDA approved standard of minimum 2D joint space width measurement (Fig. 6).

## Discussion

Previous studies looking at the prediction of total hip replacement (THR) in older adults have identified KL grade, mean patient global assessment, NSAID use, and 2D hip shape as important factors^36–38^. We show here that 3D joint space width (JSW) is significantly dependent on future THR status across the superior joint space, narrower here by up to 1 mm in individuals having a THR within the next 5 years. This result is in keeping with the known distribution of joint space narrowing from radiographic studies, but it is the first time that this has been established in 3D. These 3D measures also show greater predictive ability than traditional 2D radiographic metrics alone. Testing the dependence of future THR against the side difference revealed a similar difference in joint space distributions, but also showed that the joint space was significantly wider posteriorly, a region that is not assessable with current radiographic standards. These results demonstrates how a 3D approach can reveal hitherto unappreciated relationships between structural disease and clinical status, a link that has been strongly questioned in 2D radiographic imaging^10^.

Indeed, radiographic measures of JSW loss can only distinguish between superomedial, superolateral, and more complete superior joint space loss^21,22^. The two relevant studies we described in Section 1 performed manual measurement of radiographic film with a magnifying glass, showing that asymmetry was most common at the superolateral site^21,22^, which we can corroborate with our 3D JSW analysis results. However, our results also show that JSW change can occur in multiple directions, which JSM can demonstrate on a case-by-case basis as in Fig. S5, or across a study cohort as we have done here with SPM.

3D statistical shape modelling showed that three of the first seven acetabular surface shape modes had significant ORs for future THR (modes 1, 2, and 6). Verbal description of these shape modes is a difficult process since they can be abstract, but one overwhelming feature that JSM has revealed is the tendency for hips that have disease to have increased medial coverage, a phenomenon that can be explained by the presence of encroaching osteophytes (shape modes 2 and Fig. S5(d)), but one that has never been shown before to be of predictive value. To the best of our knowledge, only one previous study has analysed 3D hip shape using the proximal femur from MRI imaging data with an SSM to show association with other imaging features of osteoarthritis, but none have ever examined the acetabular shape in 3D, or the joint its entirety whether in 2D or 3D^39^. These shape modes may also have implications in the form of altered 3D biomechanics as a joint fails, which would be of interest to address in future studies.

Averaging all the THR acetabular surfaces compared to respective controls separately revealed differences in their shape, with future THR hips tending to be smaller, broader across the anterior and posterior regions of the joint space, and with increased coverage of the acetabular fossa (Fig. 4). It should also be considered that any relationship of scale with disease risk could arise from the strong association of shape mode 1 with sex, with females having smaller hips. These shape results are similar to those published by Agricola *et al*.^40^, who showed that significant 2D shape modes from radiographs associated with an increased risk of future THR within 5 years had an AUC of 0.81 in a predictive model. In our model, combining 3D parameters of relative minimum 3D JSW and 3D shape mode data outperformed the current 2D radiographic gold standards of KL grade and minimum 2D JSW with an AUC of 0.81 compared to 0.72 and 0.73 respectively, an improvement of 10–12%. All imaging parameters outperformed baseline hip pain (AUC 0.69), while a combination of KL grade and 3D JSM parameters achieved very good prediction of future THR within 5 years with an AUC of 0.86, 0.14 better than KL grade alone and 0.13 than minimum 2D joint space width, an improvement of 18–19%. One recent study showed that imaging factors enhanced a basic model for prediction of incident hip radiographic osteoarthritis that encompassed demographic and questionnaire data^41^, but the best AUC achieved was 0.74 for this combined model, whereas we have shown an ability to predict future THR with imaging features alone when including 3D data.

## Limitations

By using THR as an outcome in this study, we recognise that we are predicting end-stage disease in an older population that are more likely to have a joint replacement for more advanced disease than a relatively younger population. Although CT imaging was acquired in the supine position, one previous study has shown that there is unlikely to be any difference in JSW values between standing and supine acquisitions^42^. This issue is currently void for CT acquisition because current technologies do not yet acquire standing CT at the hip joint. We also recognise that standardised positioning of the hip joints was not set during image acquisition, but significant results were achieved in the retrospective analysis of prospectively collected data despite noise that uncontrolled positioning might introduce. Future studies could control for this by wrapping the knees and ankles together at the time of acquisition, with knees flat on the CT gantry. Cone beam CT (CBCT) is a well-established technique that holds promise not only for dose reduction, but also opening up further opportunity for JSM to be used in 3D biomechanical assessment of the hip joints in a standing position. We also recognise that our radiographic measures of disease were performed using digitally reconstructed radiographs of CT imaging data and were therefore not true radiographs, but these were not available from the study cohort and so we created the best available alternative, which has been used to generate radiographic scores in previously published studies^27,43^. This may lead to a slightly different interpretation of KL grade, but assessment has been shown to be reliable, and the effect would be systematic. Importantly, the registration of our joint surfaces is also an ill-posed problem, and although we know that our technique allows representation of results on a canonical surface in 3D for SPM analysis (in which we account for shape), there is no guarantee that other registration techniques might not deliver slightly different results that could affect significance in repeat regression or predictive analyses; this problem needs further attention from a morphometric perspective to seek a suitable solution, as would all shape mode analysis studies that are not underpinned by well-defined homologous landmarks.

## Conclusion

3D joint space mapping of standard clinical CT imaging results in substantially better prediction of future THR than current 2D radiographic gold standards in healthy older individuals. At best, we have been able to show an 18% improvement using 3D measures with KL grade over the FDA-approved measure of minimum 2D radiographic joint space width. This method also shows significant differences in JSW between cases and controls across regions that are undetectable using radiographic techniques by looking at JSW asymmetry, namely the posterior joint space. We have also demonstrated for the first time that 3D shape of the acetabulum has significant associations with future THR. Contrary to the message from a recent large-scale study from the Framingham and OAI cohorts^10^, this work suggests that imaging should remain relevant for hip osteoarthritis assessment when enhanced with 3D information, while also raising the important question of whether similar improvements can be achieved from the application of JSM at other joints such as the knee and ankle. This is not a claim that the technique should be expedited into clinical practice, but our opinion is that it should be further explored in many of the roles that radiographs undertake in exploratory research and clinical trials to see what further improvements can be gained. Given that JSM has already been shown to have reliability at best twice that of radiographs^20^, we have presented evidence here suggesting a need to rethink the approach to x-ray based imaging assessment of osteoarthritis to encompass 3D information. An important question is whether this can be achieved with minimal increased risk from radiation exposure that comes with CT, particularly at the hip, noting that reduced dose protocols that would be suitable for these purposes are already available^44^. CBCT holds promise for JSM, both through its reduced dose and the opportunity to investigate weightbearing lower limb joints in 3D, which could also lead to further important biomechanical insights into osteoarthritis. We believe these results advocate the use of JSM in research and epidemiological studies as an adjunct to other non-ionising radiation techniques such as MRI and ultrasound, with further exploration warranted into its utility in roles such as disease stratification, monitoring, and outcome prediction.

## Supplementary information

Supplementary tables and figures.

Average THR and non-THR shape.

Shape mode 1.

Shape mode 2.

Shape mode 3.

Shape mode 4.

Shape mode 5.

Shape mode 6.

Shape mode 7.

Canonical hip joint surfaces.

## Data Availability

• Stradwin is freely available to download from http://mi.eng.cam.ac.uk/~rwp/stradwin/. • wxRegsurf is freely available to download from http://mi.eng.cam.ac.uk/~ahg/wxRegSurf/. • The Surfstat package is freely available to download from http://www.math.mcgill.ca/keith/surfstat/surfstat.zip. • All CT imaging data are anonymised but are not publicly available due to protection of patient identity and confidentiality in accordance with the AGES-Reykjavik cohort study ethics. Reasonable requests for sharing of this data can be made to the study committee through Sigurdur Sigurdsson. • All JSM output data (acetabular object surfaces, 3D joint space width thickness and error maps, and object-to-canonical registration files) have been uploaded to the PURE University of East Anglia data repository and can be made available on request through Tom Turmezei.
