# Supplementary material for: Quantitative 3D imaging parameters improve prediction of hip osteoarthritis outcome
Source: Sci Rep. 2020 Mar 5;10:4127. doi: 10.1038/s41598-020-59977-2 (PMC7058047; doi:10.1038/s41598-020-59977-2)
Supplement: Supplementary file 1 — Supplementary tables and figures. [file 41598_2020_59977_MOESM1_ESM.pdf]

Title

Quantitative 3D imaging parameters improve prediction of hip osteoarthritis outcome

Author list and affiliations

Turmezei TD[1*], Treece GM[2], Gee AH[2], Sigurdsson S[3], Jonsson H[4], Aspelund T[3], Gudnason V[3], Poole KES[5].

1 Department of Radiology, Norfolk and Norwich University Hospital, Norwich, U.K.

2 Cambridge University Engineering Department, Cambridge, U.K.

3 Icelandic Heart Association, Kopavogur, Iceland

4 Department of Rheumatology, Landspitalinn University Hospital, Reykjavik, Iceland

5 Department of Medicine, University of Cambridge, U.K.

Supplementary Figures & Tables

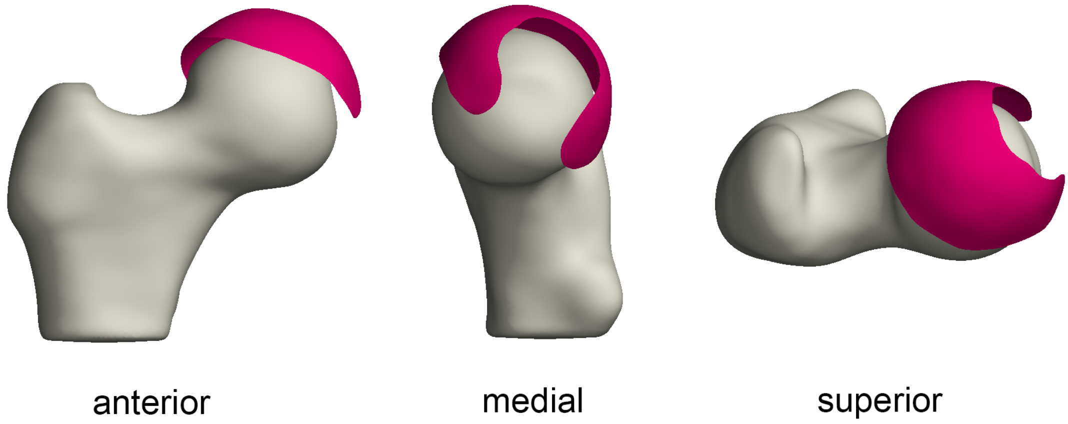

Figure S1 The canonical acetabular joint surface (pink) shown over a canonical proximal femur. This representation is also supplied as rotating 3D visualisation (canonicals_slow.gif).

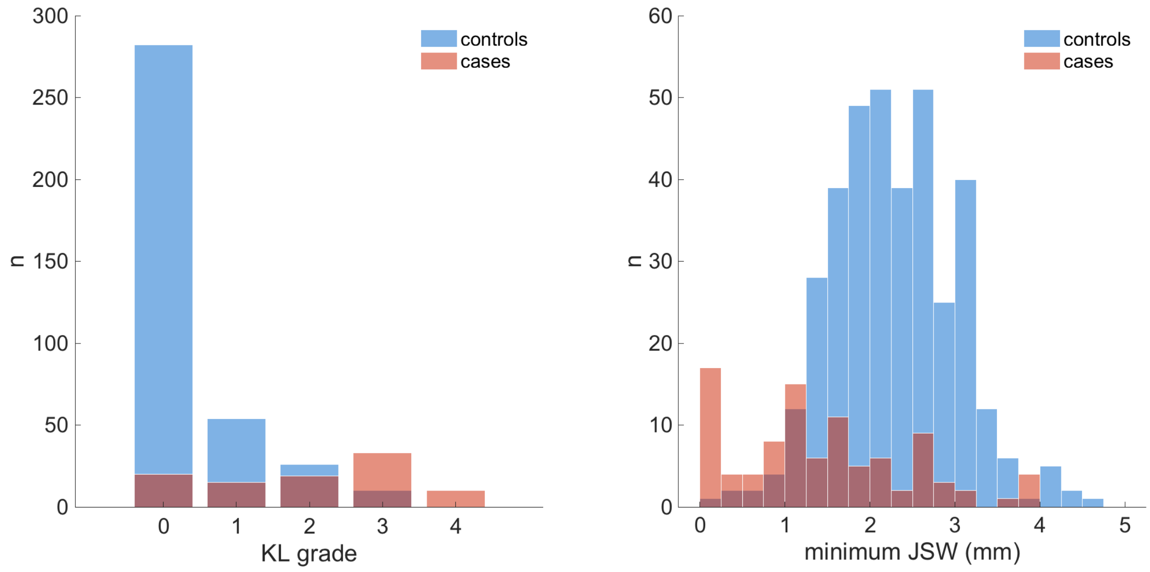

Figure S2 KL grade and minimum 2D JSW distributions for each THR case and all control hips.

KL grade	0	1	2	3	4
**THR cases**	**15**	**13**	**15**	**28**	**7**
	**(19.2)**	**(16.7)**	**(19.2)**	**(35.9)**	**(9.0)**
**All controls**	**144**	**23**	**14**	**4**	**0**
	**(77.9)**	**(12.4)**	**(7.6)**	**(2.2)**	**(0.0)**

Table S1 KL grade for each THR case and all control hips (percentage).

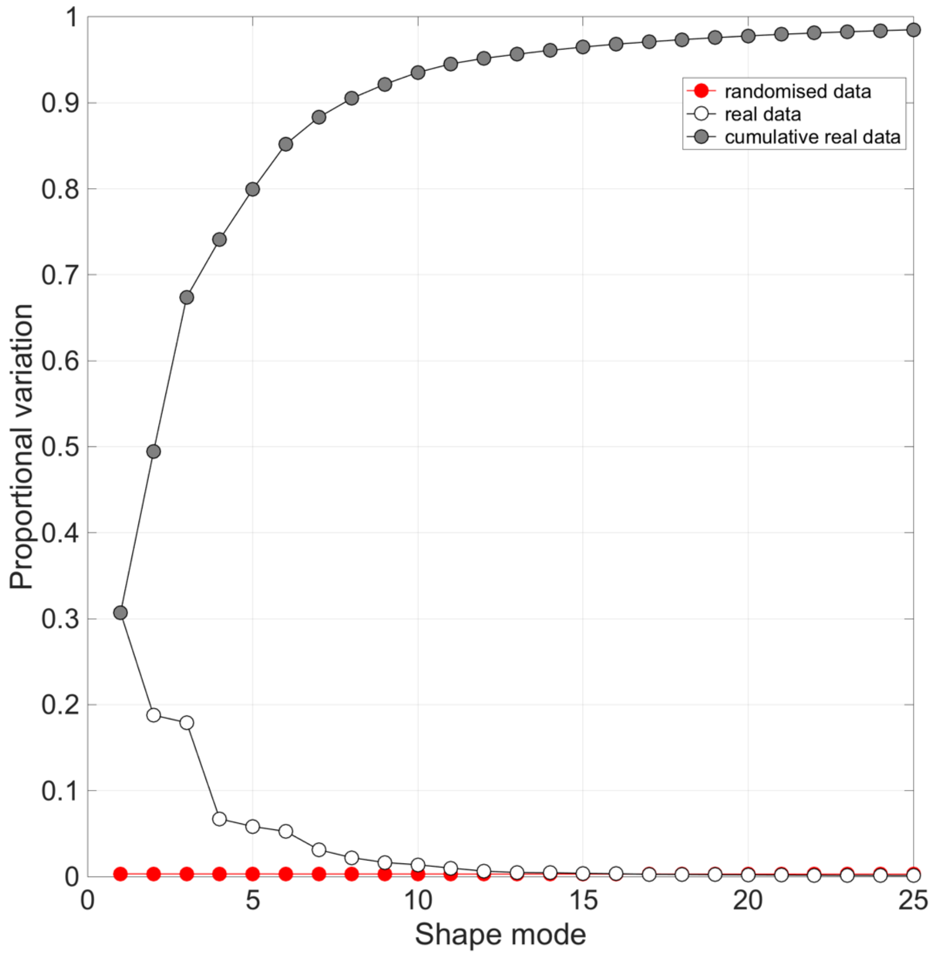

Figure S3 Proportional and cumulative proportional contributions of shape modes to overall shape in the SSM. Randomised coefficient data rerun through PCA analysis gives an idea of background noise (plotted in red). The first 16 shape modes were greater than the noise threshold, while 7 shape modes accounted for up to 90% of overall shape variation.

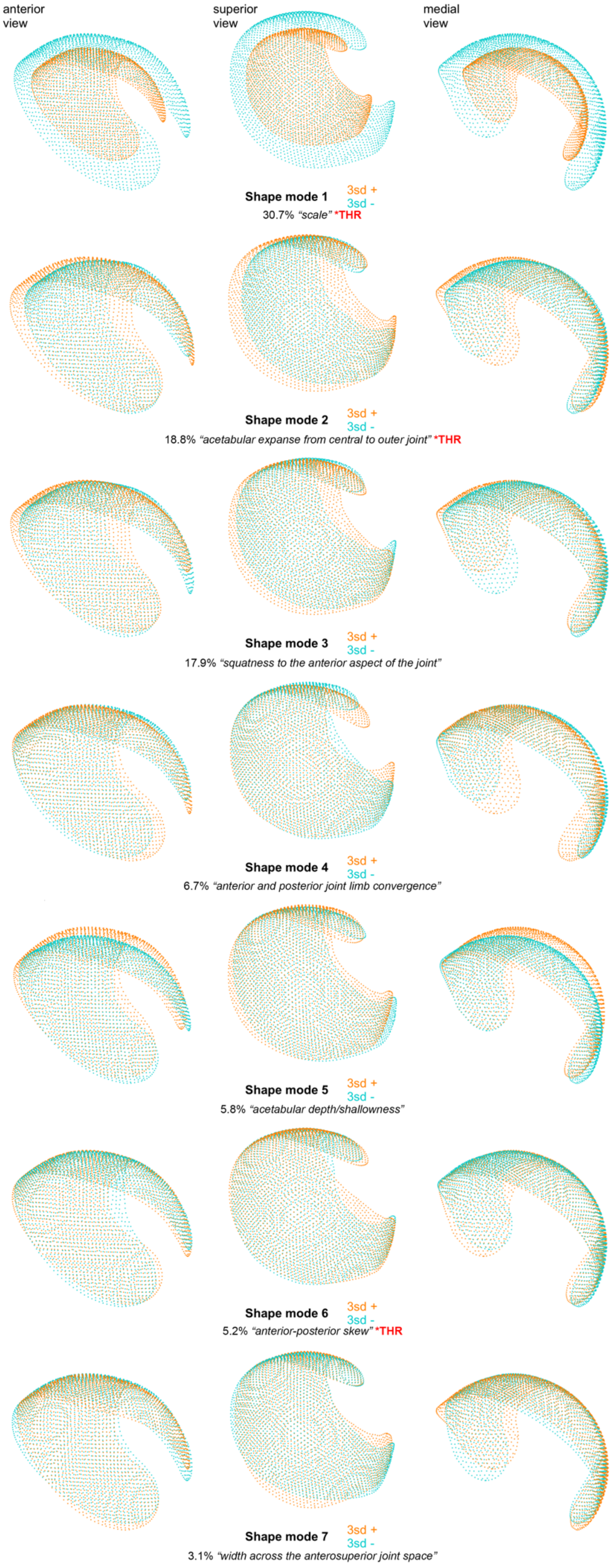

Figure S4 Results from the acetabular surface SSM. Principal component analysis of the shape mode coefficient matrix was used to determine 3D shape modes of the acetabular surface, with percentage contribution to overall shape and a verbal description given. Shape modes 1, 2, and 6 were significantly associated with future THR (red asterisk). ORs are shown in Table 2. Each shape mode is also supplied as rotating 3D visualisation (sm1.gif to sm7.gif).

	OR (± 1.96 x SE limits)	p value
Shape mode 8	1.04 (0.87 - 1.25)	0.67
Shape mode 9	1.20 (1.01 - 1.42	0.03
Shape mode 10	0.91 (0.77 - 1.08)	0.30
Shape mode 11	1.17 (1.00 - 1.37)	0.13
Shape mode 12	1.04 (0.88 - 1.23)	0.69
Shape mode 13	1.01 (0.86 - 1.18)	0.91
Shape mode 14	1.11 (0.94 - 1.30)	0.200
Shape mode 15	0.84 (0.72 - 0.99)	0.03
Shape mode 16	0.91 (0.79 - 1.05)	0.22

Table S2 Odds ratios (ORs) with ±1.96 x SE (standard error) limits for future THR for shape modes 8 to 16. No results showed significance below the conservative Bonferroni corrected threshold of $p < 0.003$.

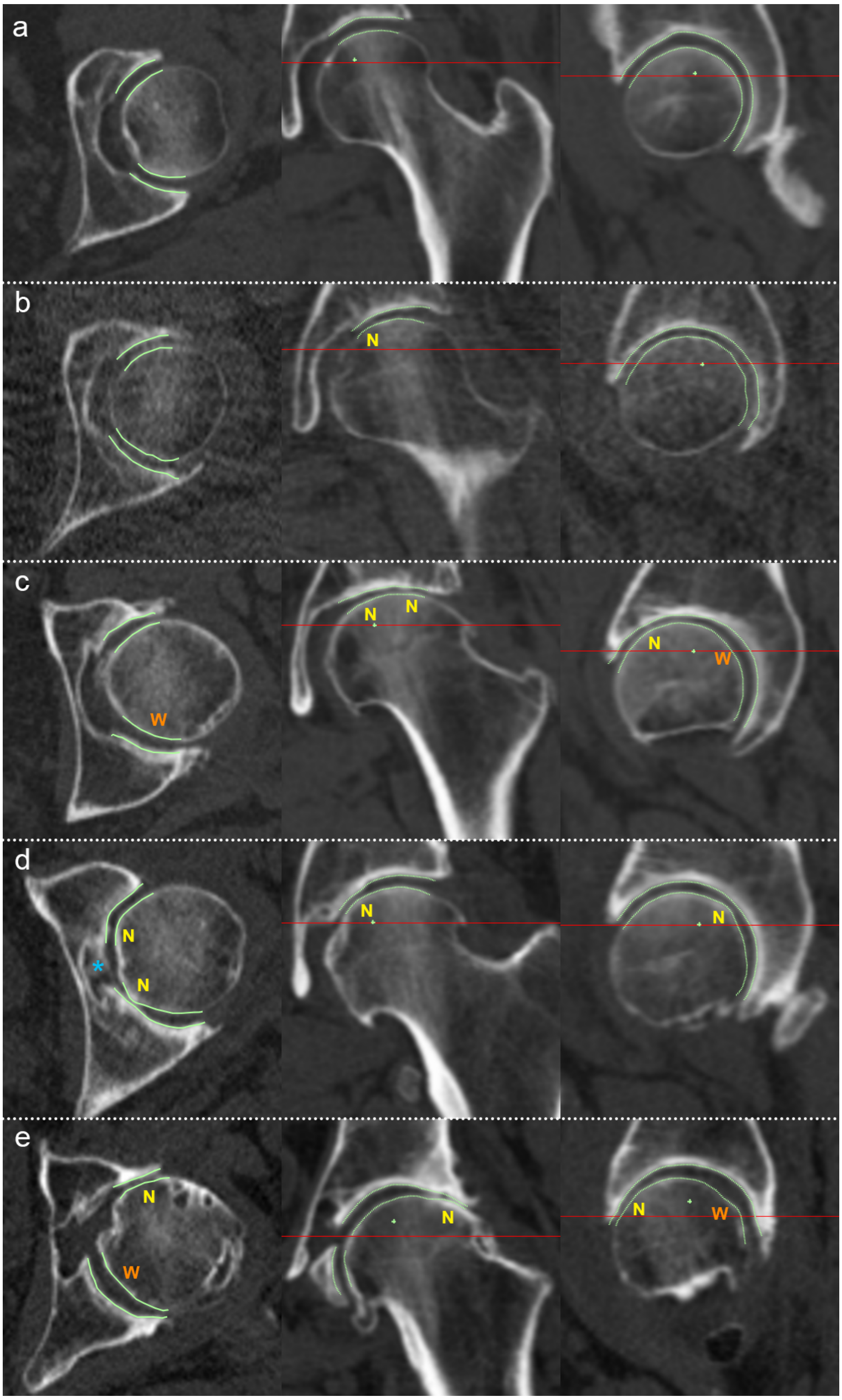

Figure S5 Directional distributions of JSW loss shown in the axial, sagittal, and coronal imaging planes (each row from left to right); the red line in the coronal and sagittal frames shows the axial frame level. Femoral and acetabular joint space margins are shown in green. **a** *"Normal":* left hip in a 74 yr old male, no THR, no pain, KL0, mJSW 3.1 mm; **b**

Superomedial narrowing: left hip in an 81 yr old female, no THR, no pain, KL0, mJSW 1.6 mm; this example also shows the robustness of the JSM, with good performance despite noise in the imaging data; **c** *Superior narrowing:* left hip in a 74 yr old male, THR, no pain, KL3, mJSW 0.5 mm; **d** *Medial narrowing:* left hip in a 72 yr old male, no THR, no pain, KL2, mJSW 0.9 mm; **e** *Anterior & superolateral narrowing, posterior widening:* right hip (flipped) in a 72 yr old female, THR, pain, KL4, mJSW 0.3 mm. N = region of narrowing; W = region of widening; * = acetabular floor osteophytes.

Supplementary Material

Files for Figure 4:

shape_thr.gif

Files for Figure S1:

canonicals_slow.gif

Files for Figure S4:

sm1.gif

sm2.gif

sm3.gif

sm4.gif

sm5.gif

sm6.gif

sm7.gif
