# Supplementary figures and images for: Quantitative 3D imaging parameters improve prediction of hip osteoarthritis outcome
Source: Sci Rep. 2020 Mar 5;10:4127. doi: 10.1038/s41598-020-59977-2 (PMC7058047; doi:10.1038/s41598-020-59977-2)

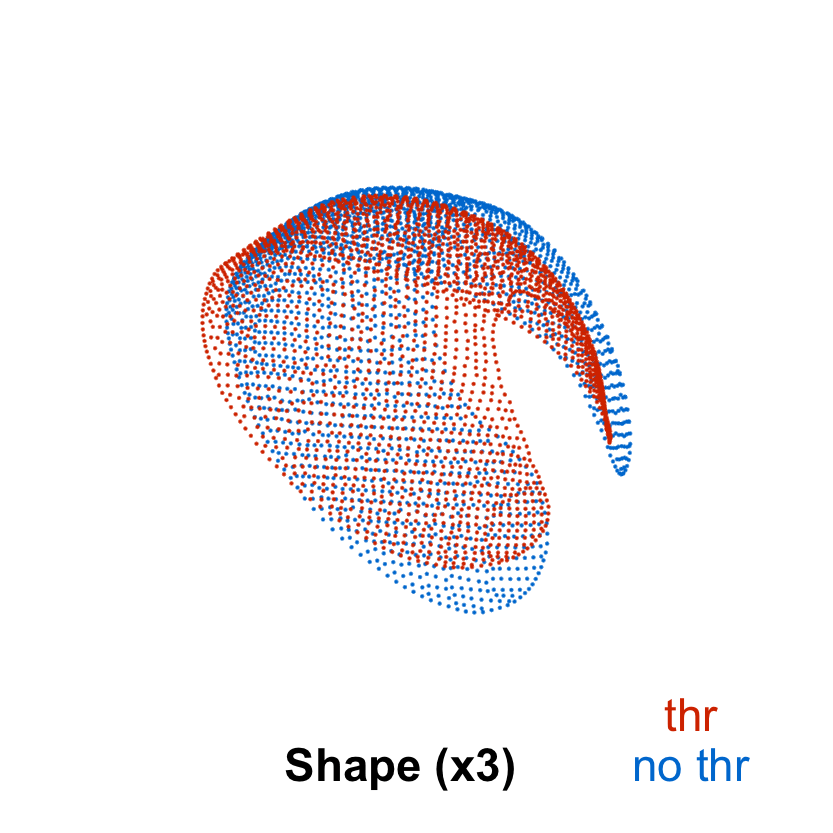

Supplement: Supplementary file 2 — Average THR and non-THR shape. [file 41598_2020_59977_MOESM2_ESM.gif]

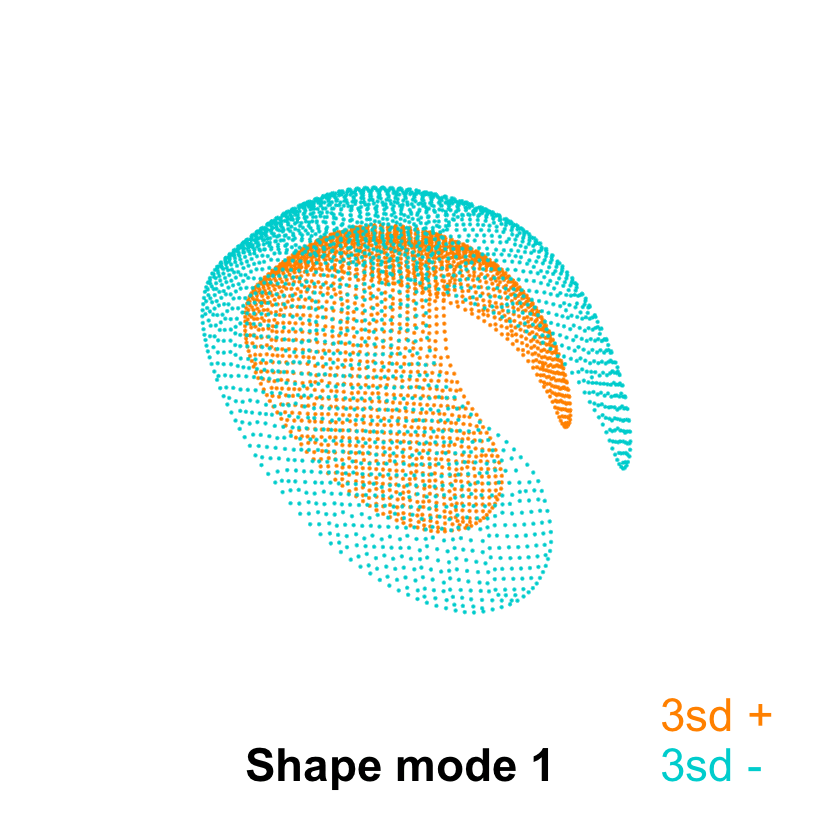

Supplement: Supplementary file 3 — Shape mode 1. [file 41598_2020_59977_MOESM3_ESM.gif]

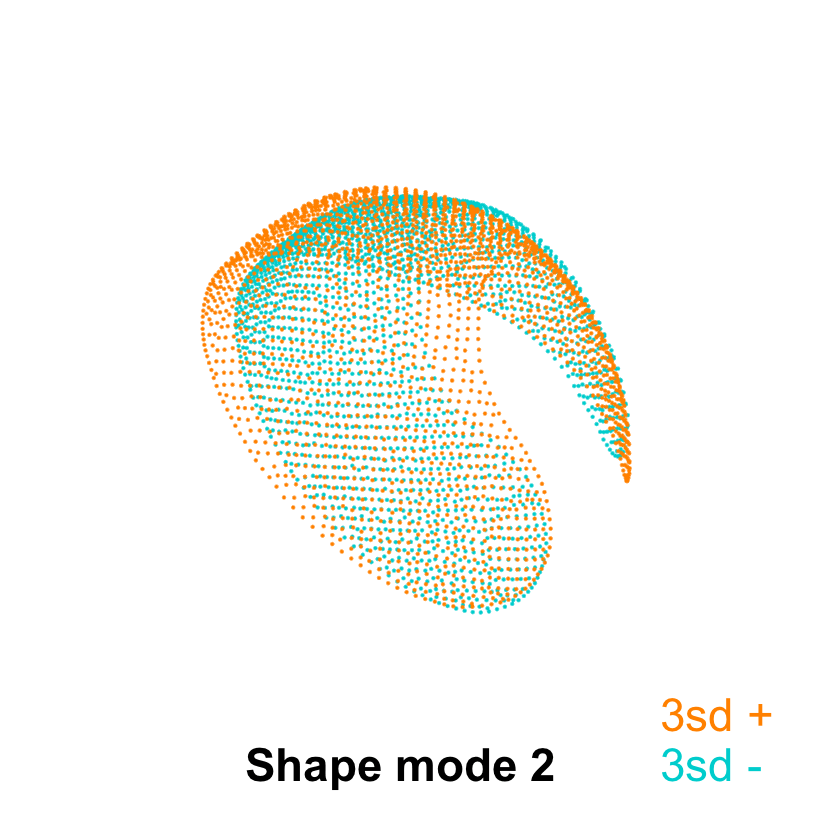

Supplement: Supplementary file 4 — Shape mode 2. [file 41598_2020_59977_MOESM4_ESM.gif]

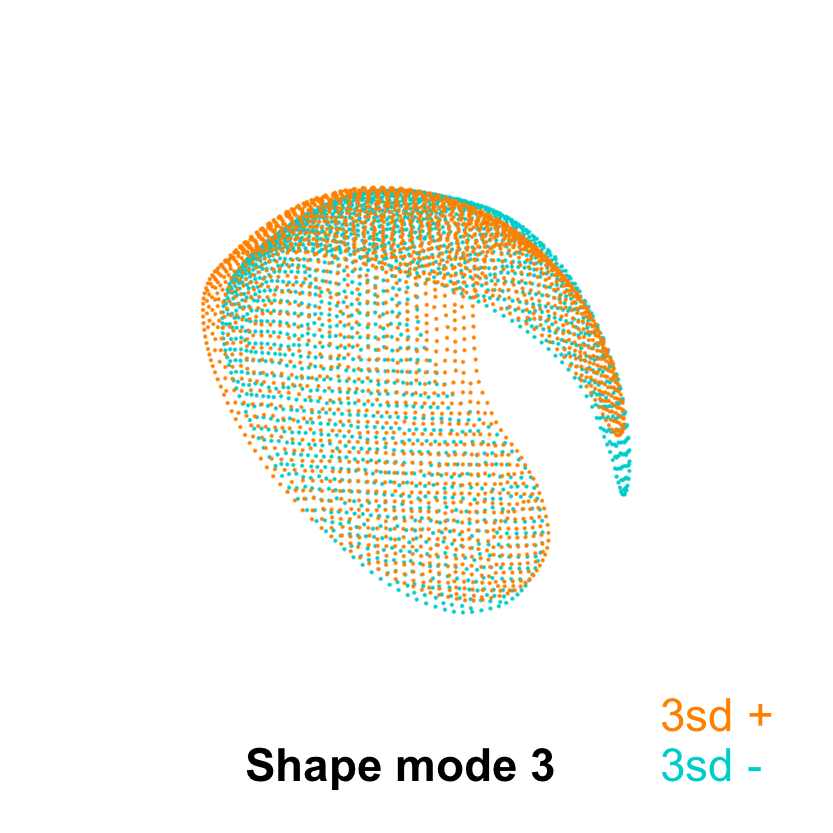

Supplement: Supplementary file 5 — Shape mode 3. [file 41598_2020_59977_MOESM5_ESM.gif]

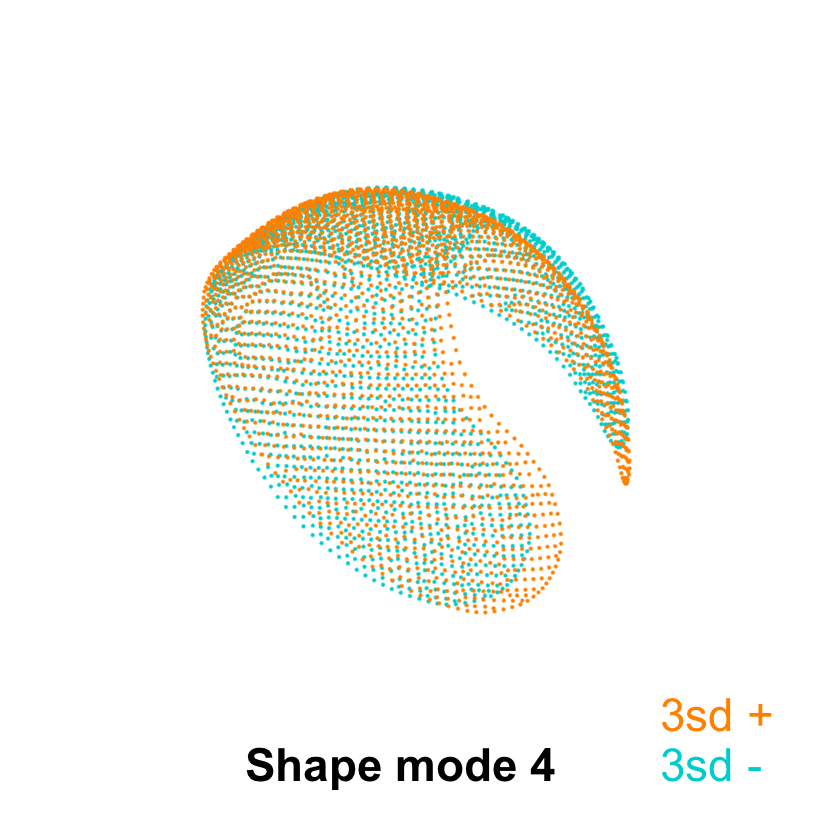

Supplement: Supplementary file 6 — Shape mode 4. [file 41598_2020_59977_MOESM6_ESM.gif]

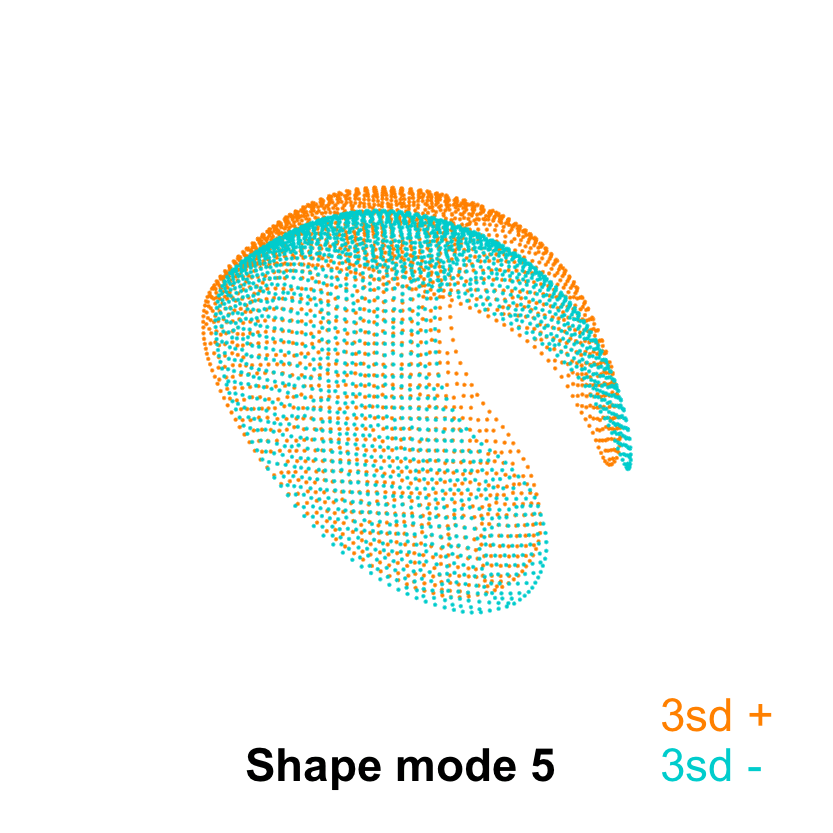

Supplement: Supplementary file 7 — Shape mode 5. [file 41598_2020_59977_MOESM7_ESM.gif]

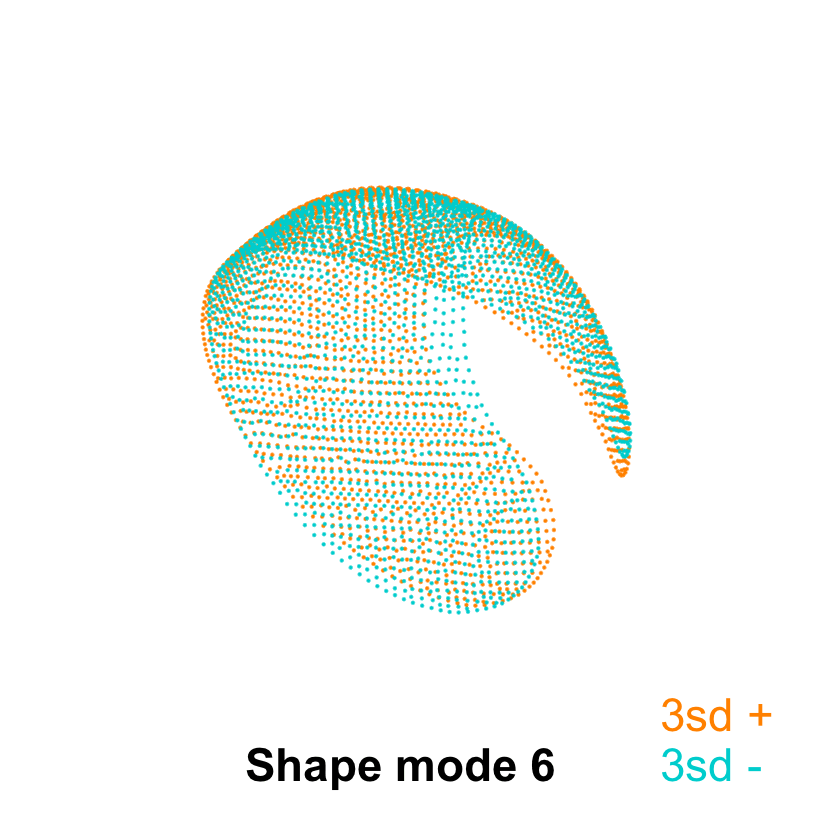

Supplement: Supplementary file 8 — Shape mode 6. [file 41598_2020_59977_MOESM8_ESM.gif]

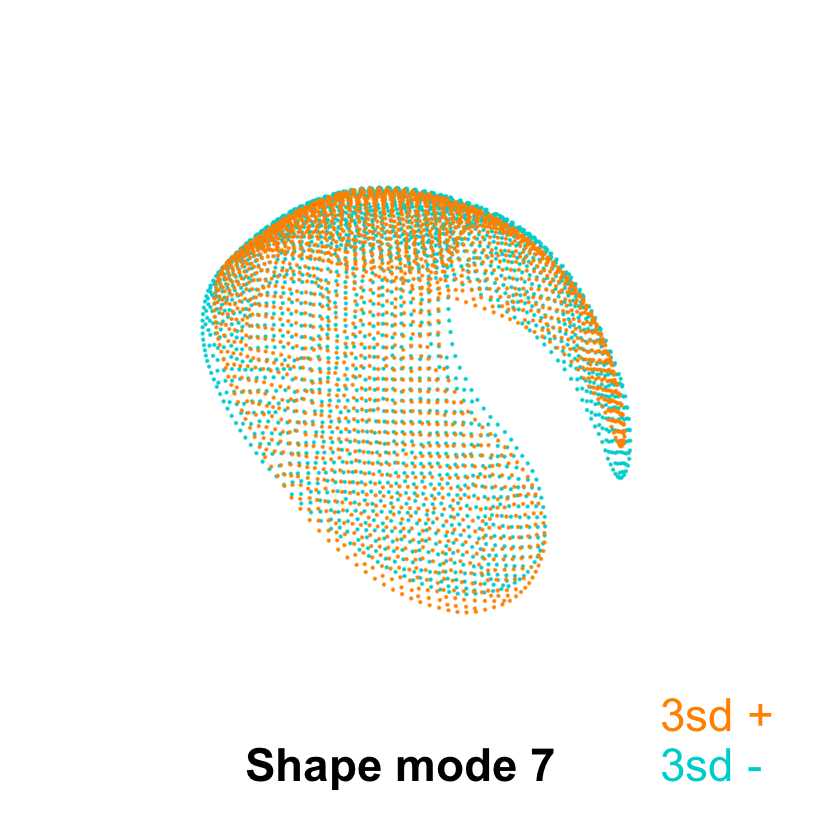

Supplement: Supplementary file 9 — Shape mode 7. [file 41598_2020_59977_MOESM9_ESM.gif]

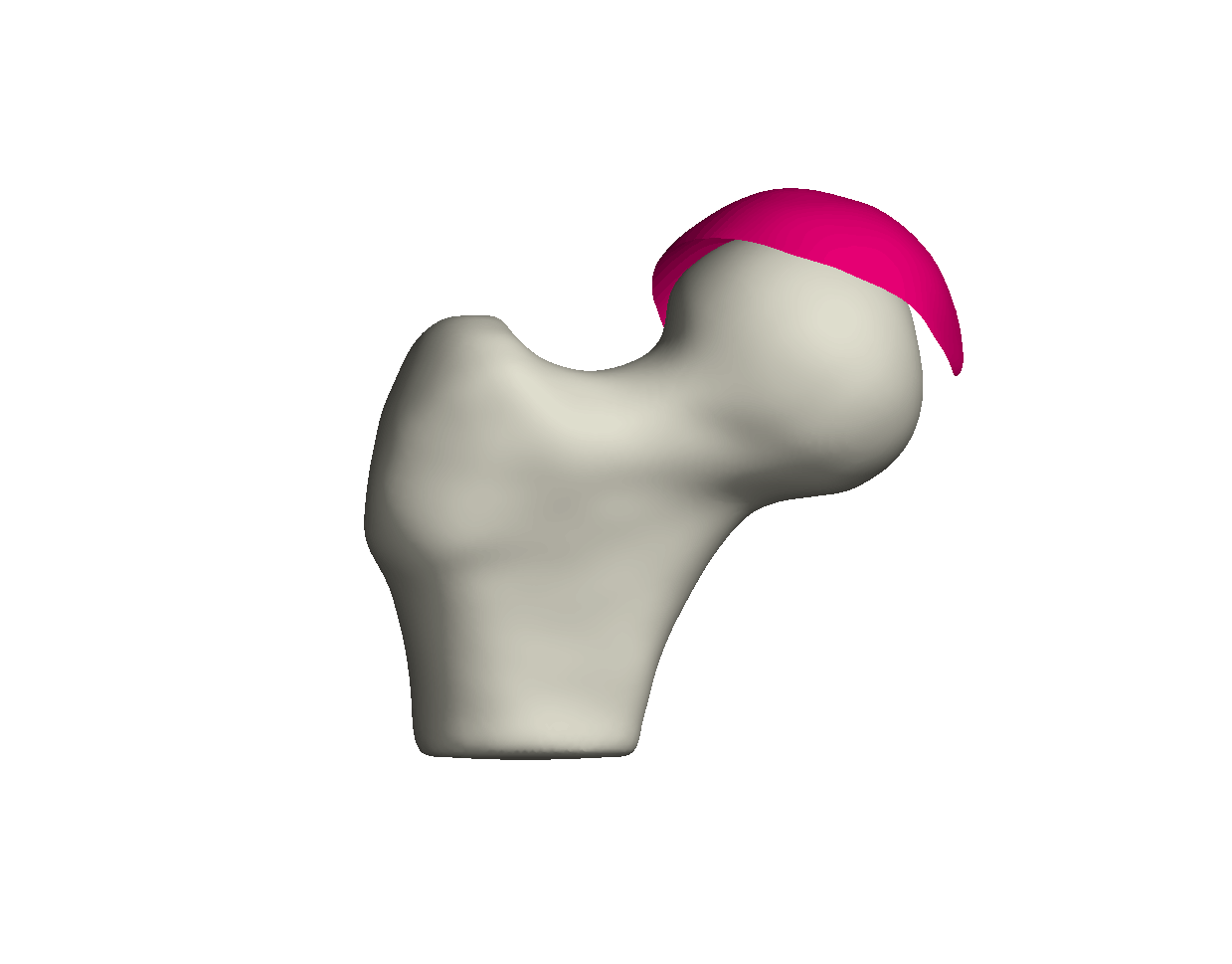

Supplement: Supplementary file 10 — Canonical hip joint surfaces. [file 41598_2020_59977_MOESM10_ESM.gif]
